# Differential vulnerability to the punishment of cocaine related behaviours: effects of locus of punishment, cocaine taking history and alternative reinforcer availability

**DOI:** 10.1007/s00213-014-3648-5

**Published:** 2014-06-21

**Authors:** Yann Pelloux, Jennifer E. Murray, Barry J. Everitt

**Affiliations:** 1 Institut de Neuroscience de la Timone, UMR 7289, CNRS Aix-Marseille Université, Marseille, France; 2 Department of Psychology and Behavioural and Clinical Neuroscience Institute, University of Cambridge, Downing Street, Cambridge, UK

**Keywords:** Cocaine seeking-taking, Alternative reinforcer, Compulsivity, Rat

## Abstract

**Background:**

The availability of alternative reinforcement has been shown to reduce drug use, but it remains unclear whether it facilitates a reduction or cessation of drug seeking or taking.

**Objectives:**

We compared the effects of punishment of cocaine seeking or taking behaviour after brief or extended cocaine-taking histories when behavioural reallocation was facilitated or not by making available an alternative ingestive reinforcer (sucrose).

**Methods:**

In the first experiment, punishment of either seeking or taking responses was introduced immediately after training on the seeking-taking chained schedule. In the second experiment, punishment of cocaine seeking was introduced after 12 additional days of either 1 or 6 h daily access to cocaine self-administration. In both experiments, beginning 1 week before the introduction of punishment, a subset of rats had concurrent nose poke access to sucrose while seeking or taking cocaine.

**Results:**

The presence of an alternative source of reinforcement markedly facilitated behavioural reallocation from punished cocaine taking after acquisition. It also facilitated punishment-induced suppression of cocaine seeking after an extensive cocaine self-administration history likely by prompting goal-directed motivational control over drug use. However, a significant proportion of rats were deemed compulsive—maintaining drug use after an extensive cocaine history despite the presence of abstinence-promoting positive and negative incentives.

**Conclusion:**

Making available an alternative reinforcer facilitates disengagement from punished cocaine use through at least two different processes but remains ineffective in a subpopulation of vulnerable animals, which continued to seek cocaine despite the aversive consequence of punishment and the presence of the alternative positive reinforcer.

## Introduction

While most individuals never go beyond “recreational” drug consumption even after prolonged use, some lose control over drug taking and develop compulsive drug use in which they persist in using the drug despite aversive consequences and at the expense of other rewards (Diagnostic and statistical manual of mental disorders, 2000). Therefore, the investigation of the psychological, neural, cellular, and molecular mechanisms underlying the transition from controlled to compulsive drug use raises the important issue of the construct validity of animal models of compulsive drug seeking.

Clinical data suggest that negative consequences directly related to drug use are a major reason for abstinence from cocaine use (Waldorf et al. 1991), and this has led to the development of novel models of abstinence based on the suppression of self-administration by an aversive contingency (Spanagel and Holter 1999; Heyne and Wolffgramm 1998; Panlilio et al. 2003; Deroche-Gamonet et al. 2004; Pelloux et al. 2007; Cooper et al. 2007) rather than by the extinction of instrumental drug-taking behaviour used in many studies of relapse and reinstatement (for review, see Shaham et al. 2003). These latter studies depend upon removal of the drug reinforcer by the experimenter which results in instrumental extinction of the taking response, a situation rarely, if ever, encountered by human drug users.

Another important factor which has been emphasised by Ahmed (2005) is that in contrast to human drug users who generally have concurrent access to a wide variety of alternative reinforcers in addition to drugs, experimental animals usually have no options in their environment other than drug reinforcement. Findings in humans with low socio-economic status, in which there are fewer opportunities for alternative forms of reinforcement, show that these individuals have higher rates of smoking (for review, see Hiscock et al. 2012) and alcoholism (Grant 1997; van Oers et al. 1999). In laboratory settings, the presence of alternative reinforcers has been shown to alter drug use in humans (for review, see Higgins 1997), in monkeys (for review, see Campbell and Carroll 2000), and in rats in both discrete choice procedures (Ahmed 2005; Lenoir et al. 2007) and under concurrent reinforcement schedules (Cosgrove et al. 2002; Kanarek et al. 1995; Klebaur et al. 2001; Mattson et al. 2001), indicating their efficacy at competing for behavioural output. Moreover, contingency management therapy has successfully been used to promote abstinence by giving addicted individuals access to alternative reinforcers for abstinence (for review, see Prendergast et al. 2006). This protocol has been shown to be the most effective psychosocial intervention for promoting abstinence and preventing relapse to cocaine addiction in individuals likely to achieve abstinence, but it does not always remain effective over extended periods of time and is ineffective in some patients (Dutra et al. 2008).

In order to understand the impact of alternative reinforcement on drug-related behaviours, we first studied the influence of making available an alternative positive reinforcer on the intermittent punishment of cocaine seeking and taking responses immediately after training on the seeking-taking task (Chen et al. 2013; Pelloux et al. 2007, 2012; Economidou et al. 2009; Jonkman et al. 2012a, b). In agreement with Konorski’s (1967) suggestion of different sources of motivational control over preparatory and consummatory behaviours (Corbit and Balleine 2003), we have shown that cocaine seeking is acquired through the formation of an action-outcome association (sensitive to incentive learning manipulations) while taking relies on more sensory-specific aspects of the reward such as associated pavlovian cues. Similarly, cocaine seeking is initially a goal-directed behaviour (Olmstead et al. 2001), but after extended training under interval schedules, it progressively becomes dominated by stimulus-response, or habitual, control (Zapata et al. 2010). We previously demonstrated that a subgroup of animals maintained cocaine seeking despite intermittent punishment only after a history of prolonged or escalated cocaine intake. These previous experiments were conducted in the presence of an alternative reinforcer (Pelloux et al. 2007, 2012), but the impact of alternative reinforcement on the ability to withhold punished cocaine use remained unclear. Consequently, we investigated the influence of an alternative reinforcer on the intermittent punishment of cocaine seeking in rats having different cocaine use histories. We hypothesised that the absence of alternative reinforcement would result in a greater propensity to seek cocaine compulsively after an extended or escalated history of cocaine reinforcement.

## Materials and methods

### Subjects

Male-outbred Lister hooded rats (Charles River, Kent, UK), weighing 180–200 g at the start of the experiment, were housed in pairs in polycarbonate cages (*L* = 40 cm, *W* = 25 cm, *H* = 18 cm) and maintained under a reversed 12-h light/dark cycle (lights on at 7.00 p.m.) at a constant temperature (21 ± 1 °C), with free access to laboratory chow (SDS) and water. The experimental procedures were conducted in accordance with the UK 1986 Animals (Scientific Procedures) Act (project licence PPL 80/1767).

### Apparatus

Instrumental training and testing took place in 12 operant conditioning chambers (29.5 × 32.5 × 23.5 cm; Med Associates, Georgia, VT) equipped with two 4-cm wide retractable levers that were mounted in the intelligence panel 12 cm apart and 8 cm from the grid floor. Above each lever was a cue light (2.5 W, 24 V), and a house light (2.5 W, 24 V) was located at the top of the opposite wall. A dipper delivered 0.04 ml of a 20 % (*w*/v) sucrose solution to a recessed magazine (3.8 cm^2^ and 5.5 cm from the grid floor) situated between the levers. Entry into this magazine was detected by the interruption of an infrared source. The floor of the chamber was covered with a metal grid with bars 1 cm apart and connected to a shock generator and scrambler (Campden Instruments, UK), which delivered 0.5-mA foot shocks. The grid was located 8 cm above an empty tray. The testing chamber was placed within sound- and light-attenuating housing equipped with a ventilation fan that also screened external noise. Silastic tubing shielded with a metal spring extended from each animal’s intravenous catheter to a liquid swivel (Stoelting, Wood Dale, IL) mounted on an arm fixed outside the operant conditioning chamber. Tygon tubing extended from the swivel to a Razel infusion pump (Semat Technical, UK) located adjacent to the housing. The operant conditioning chambers were controlled by software written in C++ using the Whisker control system (Cardinal et al. 2000).

### Surgery

Rats were anaesthetised with ketamine hydrochloride (100 mg/kg, intraperitoneal (i.p.); Ketaset) and xylazine (9 mg/kg, i.p.; Rompun) then implanted with a single catheter (CamCaths, Cambridge, UK) in the right jugular vein. The tubing ran subcutaneously over the shoulder, and the mesh end of the catheter was sutured subcutaneously on the dorsum. After surgery, rats were single housed and kept so for the rest of the experiment. To prevent infection, rats were treated post-surgically for 7 days with 10 mg/kg Baytril subcutaneously (Genus Express, Bury St. Edmunds, UK) for 7 days (Caine et al. 1992). Rats were subsequently limited to 20 g food per day provided following daily experimental sessions.

### Procedure

In experiment 1, rats were assigned to one of six groups: whether they were trained for sucrose or cocaine and whether the taking or the seeking response was punished after training. These latter groups were also distinguished by whether rats had the availability to concomitantly nose poke for sucrose thereby yielding the “sucrose taking” (*n* = 12), the “cocaine taking” (*n* = 18), the “cocaine taking + sucrose” (*n* = 16), the “sucrose seeking” (*n* = 12), “cocaine seeking” (*n* = 15) and the “cocaine seeking + sucrose” (*n* = 19) groups. In experiment 2, rats were assigned to one of four groups: whether they had short (ShA) or long (LgA) access to cocaine following seeking-taking task training and whether they had the availability to concomitantly nose poke for sucrose thereby yielding the “ShA” (*n* = 17), the “ShA + sucrose” (*n* = 31) the “LgA” (*n* = 20) and the “LgA + sucrose” (*n* = 37) groups.

#### Acquisition of the taking response

Behavioural training began 7–10 days after surgery for the cocaine groups and 1 week after arrival for the sucrose groups. For all rats, each session began with the insertion of the taking lever (left/right counterbalanced). Responding was reinforced under a fixed ratio (FR) 1 schedule. Each lever press resulted in either sucrose (0.2 ml of a 20 % sucrose solution, which was delivered by presenting the dipper five times during 5 s at the rate of one presentation per second) or cocaine (0.25-mg/kg infusion of cocaine at a rate of 0.1 ml/5 s) and was accompanied by retraction of the taking lever, offset of the house light and illumination of the stimulus light above the lever for 20 s. The sessions terminated after either 30 reinforcers or 40 min for the sucrose groups or 2 h for the cocaine groups. Training of the taking response continued for five to seven sessions.

#### Training of the seeking-taking chain

Each cycle of the seeking-taking-chained schedule started with the insertion of the seeking lever with the taking lever retracted, and the first press on the seeking lever initiated a random interval (RI) schedule. The RI parameter was progressively increased from 2 to 120 s. The first lever press after the RI had elapsed and terminated the first link of the chain, resulting in the retraction of the seeking lever and insertion of the taking lever to initiate the second link. One press on the taking lever was followed by the drug or sucrose reinforcement accompanied by the same stimulus events as during the training of the taking response. There followed a time-out period in which neither the lever was available, but the houselight was illuminated and the stimulus light was off. Thereafter, the seeking lever was reinserted to start the next cycle of the schedule. For the sucrose group, the time out was kept to 20 s, but for cocaine-trained animals, this time-out period was progressively increased across five consecutive daily sessions from 20 s to 10 min after each cocaine infusion over five consecutive sessions of training. Consequently, all the rats were responding on a heterogeneous chained (tandem FR1 RI120-s) FR1 TO schedule allowing a maximum of 11 reinforcements.

Only for experiment 2, animals were subsequently given access to cocaine over 1 h (ShA and ShA + sucrose) or 6 h (LgA and LgA + sucrose) daily for 14 sessions according to the same protocol as described for the acquisition of the taking response (i.e. no seeking component) to allow for the emergence of escalation in the LgA groups.

All eight cocaine groups received three further sessions on this seeking-taking chained schedule. During these sessions, the rats in the cocaine taking + sucrose, the cocaine seeking + sucrose, the ShA + sucrose and the LgA + sucrose groups were also trained to nose poke into the magazine for 0.04 ml of a 20 % sucrose solution, which was delivered under an RI schedule, the parameter of which was progressively increased to 60 s.

#### Punishment

All rats received a further four sessions of training under the seeking-taking chain to establish a baseline against which to assess the effects of punishment. During each punishment session, half of the cycles contained no punishment and were identical to those in baseline training. In the remaining cycles, either the taking response or the seeking response was punished: (i) punishment of taking responding, performance of the taking response delivered a .5-s foot shock rather than reinforcement for the sucrose taking, the cocaine taking and the cocaine taking + sucrose groups; (ii) punishment of seeking responding, the first response that met the RI requirement in the seeking link delivered the .5-s foot shock and led to a direct transition to the TO period without the taking link for the sucrose seeking, the cocaine seeking and the cocaine seeking + sucrose groups. The reinforced and punished cycles were presented randomly within each session for eight daily sessions of punishment (Pelloux et al. 2007). In experiment 2, only the seeking responding was punished according to the procedure described above.

#### Statistical analyses

For experiment 1, the effect of punishment of sucrose or cocaine seeking or taking was assessed by the number of cycles completed. A cycle is considered completed when cocaine is injected or shock is presented. Therefore, in all cases, this measure includes completion of the seeking cycle. A three-way mixed analyses of variance (ANOVA) between performance over the 4 days of baseline and 8 days under intermittent punishment of sucrose taking, the sucrose seeking, the cocaine taking and the cocaine seeking groups evaluated the effect of punishment, assessed by a within-subject variable of sessions and the between-subjects factors of the type of reinforcement (sucrose vs cocaine) and locus of punishment contingency (seeking vs taking). Additionally, the impact of an alternative reinforcer on punishment efficacy was evaluated through a three-way mixed ANOVA between the performance under baseline and intermittent punishment of the cocaine seeking, the cocaine taking, the cocaine seeking + sucrose and the cocaine taking + sucrose groups. Session was the within-subject factor. The between-subject variables of contingency contrasted the effects of punishment of the seeking and taking responses, whereas those of condition evaluated the effect of the availability to concomitantly nose poke for sucrose.

The effects of punishment on responding were further investigated. Within the 4 days of baseline and 8 days of punishment sessions, the total times taken to initiate the seeking link, to complete the seeking link, and to press the taking lever by the cocaine seeking, the cocaine taking, the cocaine seeking + sucrose and the cocaine taking + sucrose groups were analysed using a four-way ANOVA with contingency and condition as between-subject factors and sessions and step of the schedule as within-subject factors. Three-way ANOVAs were then performed in the cocaine taking and cocaine taking + sucrose groups with condition as the between-subject factor and sessions and step of the schedule as within-subject factors.

The impact of contingency on concomitant nose poke responding for sucrose was assessed over the 4 days of baseline and 8 days of punishment in the cocaine taking + sucrose and cocaine seeking + sucrose groups using a two-way mixed ANOVA contrasting the within-subject variable of session.

The level of investment in cocaine over sucrose-related behaviours was computed as the number of seeking or taking responses for cocaine as a proportion of the total number of motivated responses made for cocaine and sucrose across, respectively, the seeking or taking period of the schedule. This ratio was analysed in the cocaine taking + sucrose and cocaine seeking + sucrose groups through a two-way mixed ANOVA contrasting the within-subject variable of session and step of schedule and with punishment contingency as a between-subject factor.

For experiment 2, the first hour of drug self-administration was compared by mixed two-way ANOVA with session as the within-subjects factor and group as the between-subjects factor. The impact of cocaine self-administration history on baseline performance for cocaine, in the presence or absence of an alternative, was evaluated by conducting a two-way ANOVA of the averaged number of seeking responses over the 4 days of baseline with the variable of cocaine history (ShA vs LgA) and the variable of sucrose contrasting the presence or not of concomitant sucrose reinforcement. We conducted a three-way mixed ANOVA between performance over the 4 days of baseline and 8 days under intermittent punishment, assessed by a within-subject variable of session, the effect of cocaine history (ShA and LgA) and the impact of the condition of the option for alternative reinforcement. Within the ShA + sucrose and LgA + sucrose, the impact of cocaine reinforcement history on concomitant nose poke responding for sucrose was assessed over the 4 days of baseline and 8 days of punishment through two-way mixed ANOVA.

All post hoc analyses were conducted using Tukey’s honestly significant difference (HSD) tests.

## Results

### Experiment 1

**Fig. 1:**
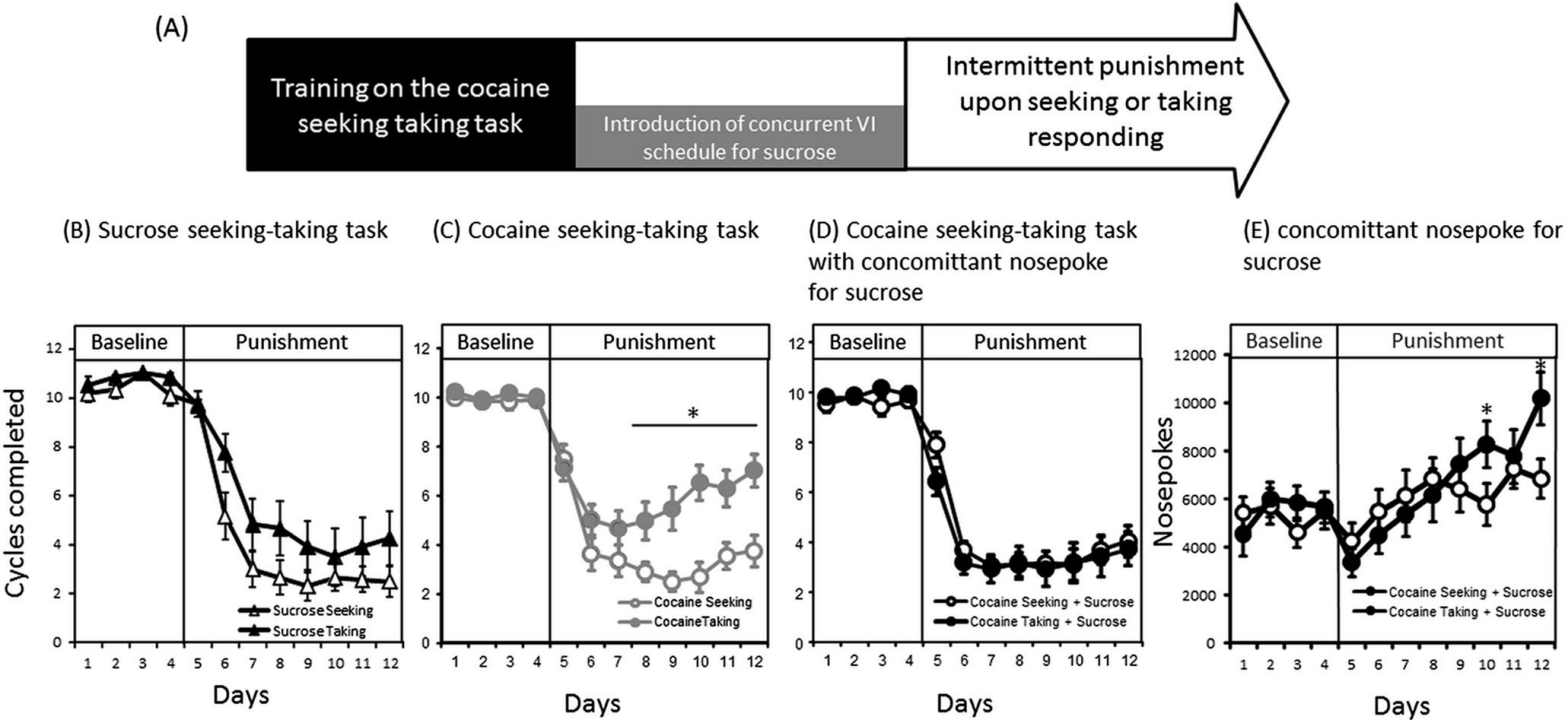
**a** The timeline of experiment 1. **b** Number of cycles completed before (*baseline*) and during punishment of sucrose taking (*black triangles*) or sucrose seeking responses after training on the sucrose seeking-taking task. **c** Number of cycles completed before (*baseline*) and during punishment of cocaine taking (*grey dots*) or cocaine seeking (*white dots*) responses after training on the cocaine seeking-taking task. **d** Number of cycles completed before (*baseline*) and during punishment of cocaine taking (*black dots*) or cocaine seeking (*white dots*) responses after training on the cocaine seeking-taking task with the availability concomitantly to nose poke for sucrose. **e** The number of nose poke responses for sucrose under baseline and punishment of the cocaine seeking or taking responses. Average ± SEM of 12 to 19 animals per group. *Tukey’s HSD; *p* < 0.05

When rats were trained with a single reinforcer (either cocaine or sucrose), all groups decreased the number of seeking cycles completed under punishment [session F(11,583) = 153; *p* < 0.0001], with the rats trained to respond for sucrose suppressing much more than those trained for cocaine [session × reinforcer F(11,583) = 8.3; *p* < 0.0001]. However, the effect of punishment significantly differed between the punishment contingencies [session × contingency F(11,583) = 4.3; *p* < 0.0001] irrespective of the reinforcer [session × reinforcer × contingency F(11,583) = 1.5; not significant (NS)]. Indeed, as illustrated in Fig. 1b, when no sucrose was available, intermittent punishment of the taking response for cocaine was less effective in suppressing responding than intermittent punishment of the seeking response (*p* = 0.002).

Among animals trained on cocaine seeking-taking (Fig. 1c, d), we again observed that all groups suppressed responding under punishment [session F(11,704) = 202; *p* < 0.0001]. However, the concomitant availability to nose poke for sucrose significantly interacted with the effect of punishment contingency [session × condition × contingency F(11,704) = 3.4; *p* = 0.0001] such that there were no differences in behavioural suppression between the two punishment contingencies (i.e. of seeking or taking responses) when sucrose was made available (Fig. 1d). Nevertheless, the increase in nose poke responding for sucrose when the punishment contingency was present during punishment [session F(11,352) = 10; *p* < 0.0001] was significantly greater when the taking response was punished compared to when punishment was applied upon of the seeking response [session × contingency F(11,352) = 3.2; *p* < 0.0001] (Fig. 1d).

**Fig. 2:**
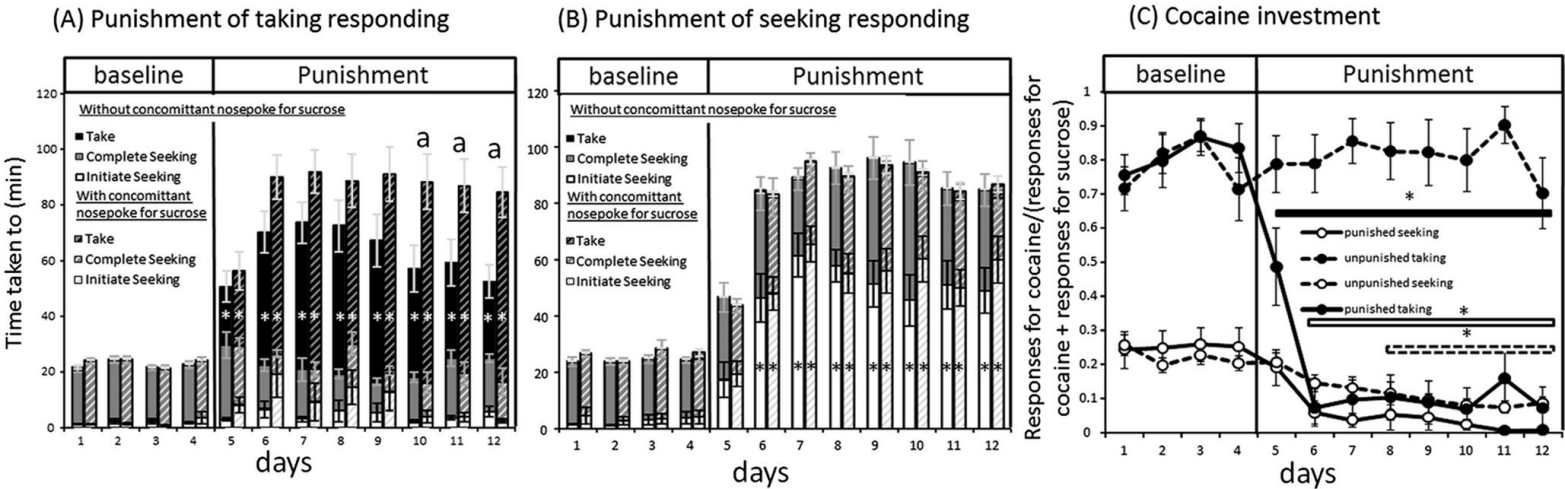
Total time per session taken to press on the taking lever (*dark histograms*) and to initiate (*light histograms*) and complete (*grey histograms*) the seeking link of the seeking-taking schedule when the taking (**a**) or the seeking (**b**) link was punished. *Solid histograms* represent the times when cocaine was the only source of reinforcement available and the *hashed histograms* when rats could concomitantly nose poke for sucrose. **c** The level of investment allocated to cocaine seeking (*white dots*) or taking (*black dots*) over motivated behaviours (see “Statistical analysesˮ section for further details) whether the cocaine responding was punished (*solid lines*) or not (*hashed lines*). Average ± SEM of 15 to 19 animals per group. *Difference with baseline, **a** differences between groups, Tukey’s HSD; *p* < 0.05

The effect of punishment on the time taken to initiate seeking, complete seeking or press the taking lever depended on whether seeking or taking behaviours were punished [F(22,1408) = 31.524, *p* < 0.001]. Indeed, intermittent punishment primarily increased latencies to initiate the punished link of the schedule. When the seeking link was punished, the latencies to initiate seeking were increased compared to baseline for sessions 6–12 (HSD; *p* < 0.05) (Fig. 2b). When the taking link was punished, the latency to take was increased for all punished sessions (*p* < 0.05) (Fig. 2a).

In animals with punished taking, there was a significant main effect of the presence of alternative reinforcement [F(1,64) = 4.673, *p* = 0.034] with the availability of sucrose resulting in increased overall latencies. The effect was most evident on the latency to respond on the taking lever [F(22,704) = 2.551, *p* < 0.001]. The time taken to press the taking lever remained higher in rats given an option for alternative reinforcement compared to rats not given this opportunity on sessions 10, 11 and 12 (*p* < 0.05) (Fig. 2a).

Investment in cocaine over sucrose-related behaviours was computed as the ratio of seeking or taking responses for cocaine: the total number of motivated responses (i.e. for both cocaine and sucrose) across the seeking or taking period of the schedule. It was revealed that cocaine was consistently taken at the expense of sucrose responding, unlike cocaine seeking responding [F(1,352) = 133; *p* < 0.001]. Intermittent punishment of cocaine seeking responding reduced the allocation of behaviour to seeking responses from session 6 onward (HSD; *p* < 0.05), but did not affect the allocation of behaviour to taking responses. In contrast, intermittent punishment of cocaine taking responses reduced both the allocation of behaviour to seeking from session 8 (*p* < 0.05) onward and to taking responses from session 4 onward (*p* < 0.05). In this latter case, the rats went from taking cocaine almost exclusively during baseline to respond predominantly for sucrose rather than cocaine when cocaine taking responses were punished (Fig. 2c).

### Experiment 2

**Fig. 3:**
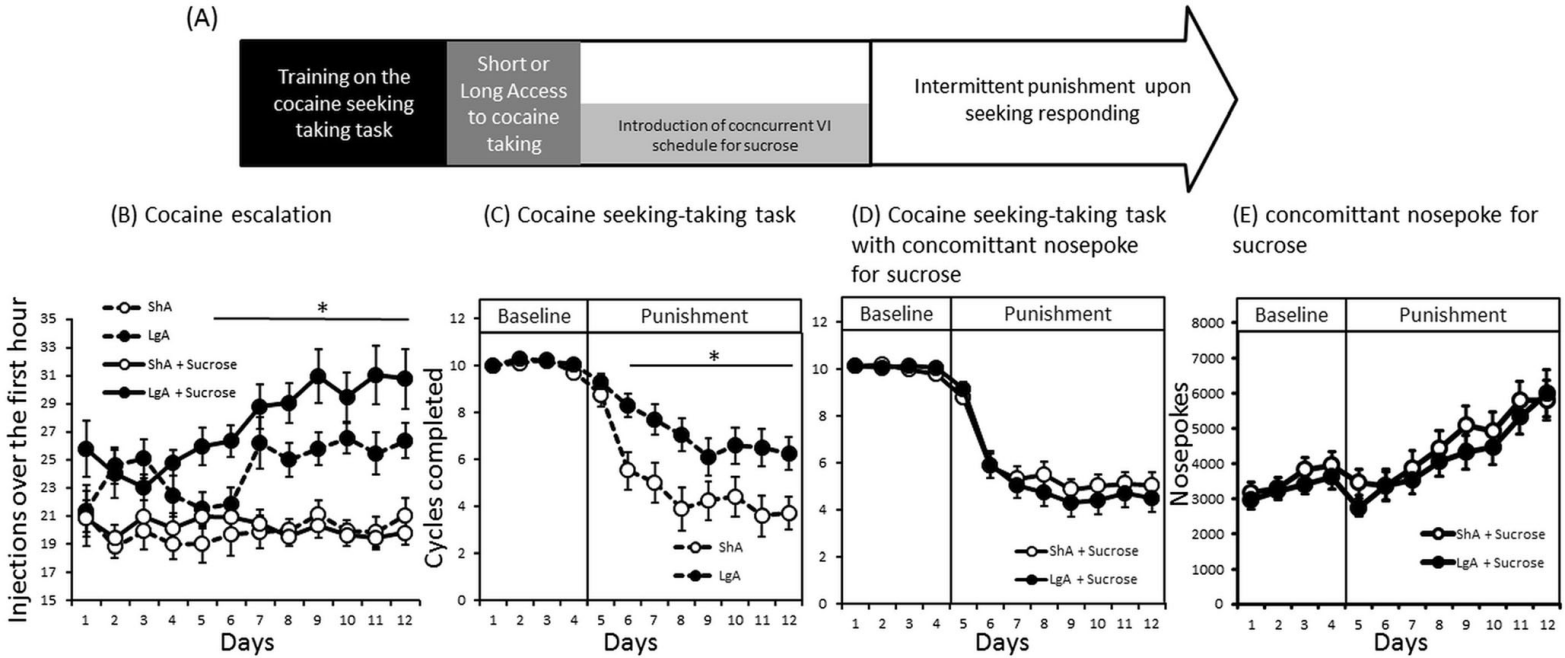
**a** The timeline of experiment 2. Early loading phase (first hour) during free access sessions (**b**). Number of cycles completed before (*baseline*) and during punishment of cocaine seeking responding after 12 days of 1 h (ShA *white dots*) or 6 h (LgA *black dots*) cocaine access without (**c**) or with (**d**) the availability of sucrose following a nose poke response. **e** The number of nose poke responses for sucrose under baseline and punishment of cocaine seeking. Average ± SEM of 17 to 37 animals per group. *Differences between groups, Tukey’s HSD; *p* < 0.05, *p* < 0.05

During the short- or long-access cocaine self-administration phase, the time of drug availability differentially affected drug intake during the first hour [session × history F(11,1111) = 3.8; *p* < 0.0001] independently of the future condition animals would experience [session × history × condition F(11,111) = 1.5; NS] (Fig. 3a). The amount of cocaine self-administered was similar in all groups during the first session [F(3,101) = 2; NS] whereas rats with only 1 h daily access to cocaine (ShA) showed a stable and controlled pattern of consumption across sessions (*F* < 1), and daily access to 6 h of cocaine (LgA) resulted in a gradual escalation across sessions during their first hour [F(11,605) = 14; *p* < 0.001] regardless of the condition they would experience subsequently [session × condition F(11,605) = 1.6; NS] (Fig. 3a).

Rats with a history of LgA completed a significantly higher number of seeking responses during seeking-taking baseline sessions compared to ShA rats [F(1,101) = 12.4, *p* = 0.04] independent of the availability of sucrose following a nose poke (*F* < 1) (data not shown).

All groups significantly reduced responding for cocaine under punishment of the seeking response [session F(11,1111) = 147; *p* < 0.0001], but the effect of punishment on the number of cycles completed significantly interacted with both the drug self-administration history and the availability of sucrose [session × history × condition F(11,1111) = 4, *p* < 0.0001] (Fig. 3b, c). When no sucrose was available, punishment-induced suppression was less pronounced in animals with an extended drug self-administration history than in animals with a more restricted cocaine history (Tukey’s HSD *p* < 0.05) (Fig. 3b). However, no differences in behavioural suppression were observed between ShA and LgA rats when sucrose was made available (*p* > 0.48) (Fig. 3c). Nose poke responding for sucrose increased when the cocaine seeking responses were punished [session F(11,726) = 25; *p* < 0.0001] and did so independently of the cocaine self-administration history (session × history *F* < 1) (Fig. 3d).

**Fig. 4:**
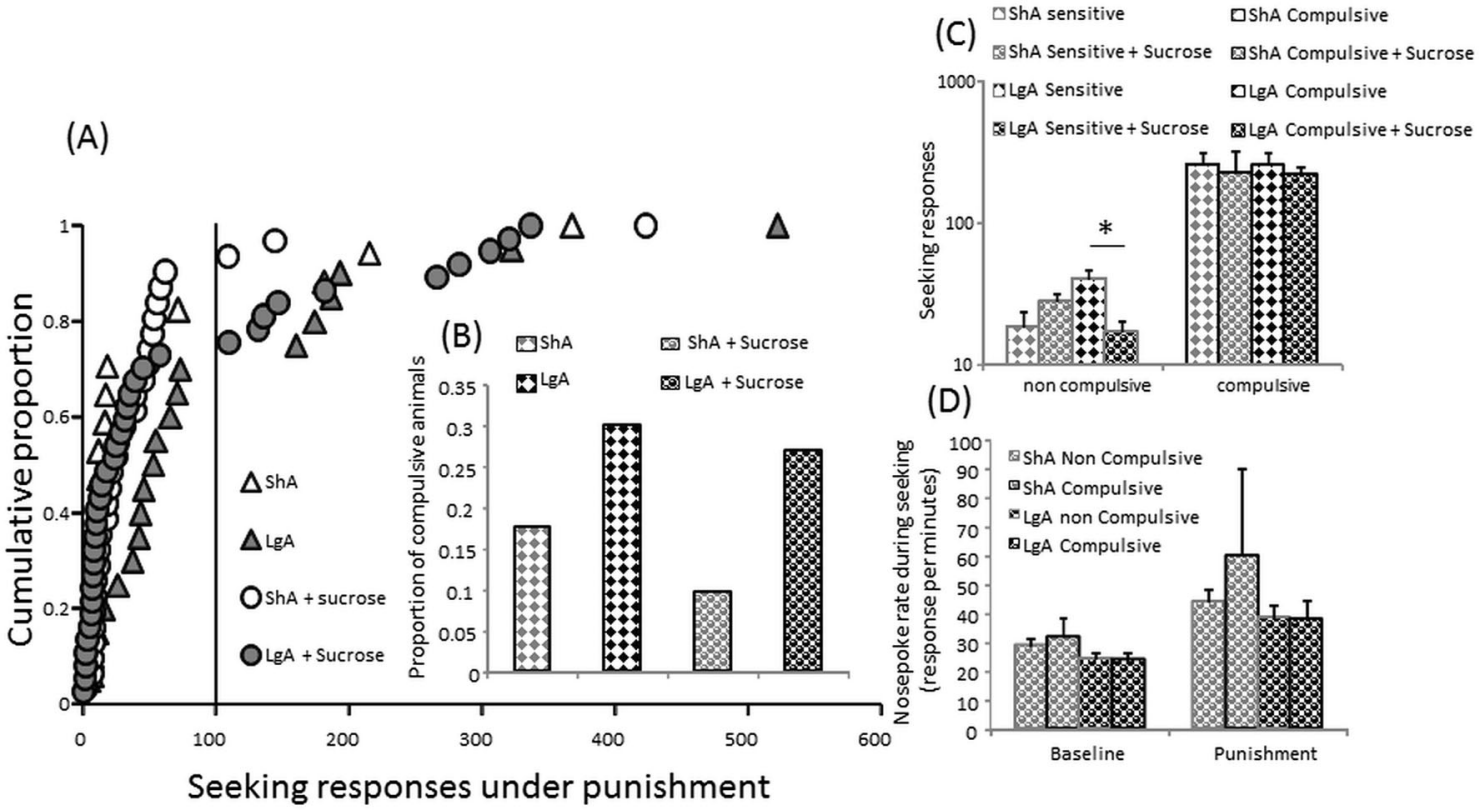
**a** Distributions of the mean number of seeking responses across the four last days of intermittent punishment of cocaine seeking responding in the “ShA” (*white triangle*), “LgA” (*grey triangle*), “ShA + sucrose” (*white circle*) and “LgA + sucrose” (*grey circle*) groups. **b** Proportion of compulsive animals in the ShA (*grey histograms*), “LgA” (*black histogram*), “ShA + sucrose” (*waved grey histograms*) and “LgA + sucrose” (*waved black histograms*) groups. **c** Mean number of seeking responses per session or **d** nose poke rate while seeking across the last 4 days of intermittent punishment of cocaine seeking responding in non-compulsive (*grey borders*) and compulsive (*black borders*) and after limited (*grey histograms*) or extended/escalated (*black histograms*) cocaine self-administration history. In **c** *diamond*-*filled histograms* represent the mean for the animals without (*diamond histograms*) and the *circle*-*filled histogram* with the availability to concomitantly nose poke for sucrose. Average ± SEM of 3 to 27 animals per group. *Tukey’s HSD; *p* < 0.05

The apparent lack of a difference between ShA + sucrose and LgA + sucrose groups in the number of cycles completed obscured an important variation in the distribution of the population of rats (Kolmogoroff-Smirnoff *Z* = 1.51, *p* = 0.021). Whereas the distribution of the ShA + sucrose group was not significantly different from a normal distribution, the distribution of the LgA + sucrose group significantly differed from a normal or lognormal distribution (Wilks-Shapiro’s *W* = 0.824, *p* < 0.001; *W* = 0.916, *p* < 0.001, respectively). Thus, in contrast to the relative homogeneity within the ShA + sucrose group, rats in the LgA + sucrose group appeared to fall into two subgroups, one sensitive to punishment and one resistant to punishment as we have reported previously (Pelloux et al. 2007). Investigation of the distributions of seeking performance across the last 4 days of punishment within the ShA, the ShA + sucrose, the LgA and LgA + sucrose revealed that all distributions were skewed with only a minority of rats performing over 100 seeking responses per session (Fig. 4a). The proportion of these compulsive animals was higher in animals with an escalated/prolonged cocaine history (Khi^2^(1) = 3.82, *p* = 0.05) with the presence of an alternative reinforcer having no impact on these proportions (Fig. 4b). Nevertheless, the presence of an alternative reinforcer reduced seeking performance in punishment-sensitive animals after extended/escalated cocaine intake [interaction history × alternative F(1,79) = 13; *p* < 0.001; HSD *p* < 0.05] (Fig. 4c).

The level of seeking responding for cocaine under intermittent punishment was unrelated to the level of unpunished responding for sucrose as all groups having access to sucrose as an alternative reinforcer increased their nose poke responding during punishment [phase F(1,64) = 24; *p* < 0.001] at similar level [interaction *F* < 1] (Fig. 4d).

## Discussion

These studies demonstrate that the availability of an alternative source of reinforcement promotes suppression of cocaine use when there is an aversive consequence or seeking or taking cocaine. The concomitant availability of a nose poke action for sucrose reinforcement enabled rats to withhold their intermittently punished cocaine-taking responses after a limited cocaine use history and also from their intermittently punished cocaine-seeking responses after an extensive cocaine self-administration history. The intermittent and unpredictable punishment of preparatory (seeking) or consummatory (taking) responses were not equally effective in producing the withholding of responses, when cocaine was the only source of reinforcement.

Previous studies comparing the effectiveness of seeking versus taking punishment have yielded inconsistent results. Whereas our present results agree with those of Church (1969) showing that instrumental response punishment more effectively suppresses behaviour than consummatory response punishment and De Costa and Ayres (1971) showing that instrumental responses are more sensitive to conditioned suppression than consummatory responses, they differ from the results of other studies. Thus, Myer (1973) showed no difference between consummatory and instrumental punishment in their overall effectiveness in suppressing responding. In contrast, Bertsch (1972) showed that consummatory response punishment was more capable of suppressing behaviour than instrumental punishment. Furthermore, Feirstein and Miller (1963) observed that the potency of consummatory or instrumental punishment in suppressing behaviour depended on whether introduction of the shock contingency was progressive or not.

These seemingly contradictory results could primarily be due to methodological differences in the procedures used such as the amount of instrumental training or the extent to which the subject could reallocate its behaviour. Indeed, the ability concomitantly to nose poke for sucrose equalised the suppression efficacy of the two punishment contingencies. These results are, however, in line with several studies showing that the opportunity to engage in alternate behaviours for either the same or different rewards leads to a greater suppression evoked by punishment than is the case when only a single response-reinforcer option is available (Thompson et al. 1999; for review, see Azrin and Holz 1966).

Punishment in a multi-task setting has consistently been shown to lead to both a decrease in the punished response and an increase in alternative responses, resulting in effective behavioural reallocation in rats (Dunham 1971), pigeons (Azrin and Holz 1966) and humans (Thompson et al. 1999). It has thus been proposed that any behaviour other than the one that is punished is reinforced by competing with the punished behaviour and thereby preventing the occurrence of punishment (Dunham 1971). Notably, we observed that punishment of cocaine responding was accompanied by an increase in the responding for the competing sucrose reinforcement indicating disengagement from punished cocaine taking was greatly promoted by the presence of this alternative likely by providing a strategy to avoid punishment. Hence, the present data support the view that the reduced ability of punishment of the taking response to suppress responding may be due to a diminished capacity to reallocate behaviour.

Similarly, we have observed that the availability of an alternative reinforcer can also counteract the effect of an extended, or escalated, cocaine-taking history on the punishment-induced suppression of drug use. Without the availability of an alternative ingestive reinforcer, punishment-induced suppression is attenuated following an extended cocaine history. However, when allowing access to sucrose, cocaine-escalated rats suppressed their cocaine seeking as much as non-escalated rats. Despite the presence of an alternative reinforcer, a subgroup of escalated rats nevertheless maintained responding for cocaine despite punishment, thereby behaving compulsively (Pelloux et al. 2007, 2012). It is unclear, though, whether the reduced ability to refrain from cocaine seeking after an extended-escalated cocaine-taking history originated from a reduced capacity to reallocate behaviour in this vulnerable subgroup. There was neither difference in sucrose-maintained responding between punishment resistant and sensitive rats during either baseline or the last 4 days of punishment nor the increase in responding for sucrose concomitant with seeking punishment dependent on the cocaine reinforcement history. Our data provided no evidence to support the involvement of altered behavioural reallocation processes in compulsive animals. Further studies are warranted to understand the psychological processes underlying punishment-induced suppression and to reveal the dysfunctional processes underlying resistance to punishment of cocaine-related behaviours. As compulsive animals maintained their cocaine seeking despite punishment and the availability of an alternative reinforcer, understanding how the presence of an alternative reinforcer alters the effectiveness of punishment may shed light on the altered processes involved in compulsive drug seeking.

Some clues may be found in the fact that animals with an extended cocaine taking history have more difficulty in suppressing cocaine seeking despite intermittent punishment, so long as cocaine is the only source of reinforcement. It has been shown that repeated amphetamine treatment accelerates the formation of stimulus-response habits in rats (Nelson and Killcross 2006; Nordquist et al. 2007). It is possible, therefore, that in our experiments, repeated cocaine reinforcement may have favoured the motivational control over seeking performance to shift and to depend to a lesser extent on the mental representation of its consequence and therefore to be less sensitive to punishment. We observed that the allocation of behaviour to seeking responses was decreased when the taking response was intermittently punished, indicating the goal-directed nature of the seeking response. The finding that punishment of the seeking response, an action that initially depends upon the value of the outcome, is more effective than punishment of the taking response, which is less sensitive to incentive learning processes and more dependent on the sensory-specific features of the reward such as associated cues (Corbit and Balleine 2003) the latter acting without the mental representation of the outcome (Holland 2004). This suggests that punishment has a more disruptive effect on actions dependent on outcome value than on actions that do not. This hypothesis is supported by data showing that inactivation of the dorsolateral striatum both reduced performance of a well-established cocaine-seeking habit that was insensitive to reinforcer devaluation (Zapata et al. 2010) and also compulsive cocaine seeking observed after an extended cocaine history (Jonkman et al. 2012b).

The opportunity to choose between responses that yield different outcomes, as in the present study, prevents the development of habitual responding (Kosaki and Dickinson 2010). Therefore, it is likely that introducing an alternative reinforcer after an extended and escalated cocaine reinforcement history may have shifted the motivational control over cocaine seeking from a habitual to a goal-directed process, thereby increasing its susceptibility to suppression when facing adverse consequences in most subjects. The inability to abstain from seeking cocaine in compulsive animals could therefore originate from the incapacity to shift from habitual to goal-directed motivational control over seeking, and this may underlie some of the difficulty in achieving abstinence in individuals that have a history of compulsive cocaine seeking.

In summary, the present results demonstrated that the presence of an alternative source of reinforcement successfully facilitated punishment-induced suppression of cocaine use. These data indicate that alternative reinforcement can facilitate response reallocation from cocaine taking after a limited cocaine history. In contrast, after an extended cocaine reinforcement history, the presence of an alternative reinforcer facilitates punishment-induced suppression of cocaine seeking; perhaps, we suggest promoting goal-directed motivational control over that behaviour. Understanding the psychological processes underlying the effect of an alternative reinforcer to promote a reduction in, or perhaps eventual cessation of, drug consumption may shed some light on the nature of the impairments in individuals unable to do so, who instead maintain their drug use despite the presence of both positive and negative incentives capable of preventing the majority of individuals from becoming addicts.
